# MicroRNAs Regulate Mitochondrial Function in Cerebral Ischemia-Reperfusion Injury

**DOI:** 10.3390/ijms161024895

**Published:** 2015-10-20

**Authors:** Yue Hu, Hao Deng, Shixin Xu, Junping Zhang

**Affiliations:** 1 Graduate School, Tianjin University of Traditional Chinese Medicine, 312 An Shan Xi Road, Nan Kai District, Tianjin 300193, China; E-Mail: tingqianliu90@sina.com; 2 Medical Experiment Center, First Teaching Hospital of Tianjin University of Traditional Chinese Medicine, 314 An Shan Xi Road, Nan Kai District, Tianjin 300193, China; E-Mail: LJdenghao@yahoo.com

**Keywords:** ischemic-reperfusion, mitochondria, miRNAs, cerebral

## Abstract

Cerebral ischemia-reperfusion injury involves multiple independently fatal terminal pathways in the mitochondria. These pathways include the reactive oxygen species (ROS) generation caused by changes in mitochondrial membrane potential and calcium overload, resulting in apoptosis via cytochrome c (Cyt c) release. In addition, numerous microRNAs are associated with the overall process. In this review, we first briefly summarize the mitochondrial changes in cerebral ischemia-reperfusion and then describe the possible molecular mechanism of miRNA-regulated mitochondrial function, which likely includes oxidative stress and energy metabolism, as well as apoptosis. On the basis of the preceding analysis, we conclude that studies of microRNAs that regulate mitochondrial function will expedite the development of treatments for cerebral ischemia-reperfusion injury.

## 1. Introduction

With substantial incidence, prevalence, and mortality [1], stroke is the second leading cause of death worldwide [2]. Ischemic strokes are caused by cerebral thrombosis or embolism within a blood vessel. As a result, local brain tissue become necrotic and apoptotic often leading to corresponding neurological deficits. One of the most effective strategies to treat this disease is immediate restoration of blood flow to the brain, although it imposes risks of further cellular necrosis and neural damage [3,4]. This damage mainly involves free radical generation, intracellular calcium overload, energy metabolism dysfunction, and apoptosis [5,6,7,8,9,10,11].

Mitochondria maintain homeostasis through energy production, synthesis of many compounds, and participation in cell signaling networks [12]. Detailed observations of the mechanisms of ischemia-reperfusion injury are difficult to conduct in the intact brain; however, cerebral ischemia may directly cause mitochondrial dysfunctions because the brain is highly susceptible to ischemic hypoxia [13]. The responses of mitochondria to cerebral ischemic-reperfusion injury are not yet completely identified, although several studies have suggested that during ischemic-reperfusion, mitochondria overproduce ROS that consume antioxidants, suppress the endogenous antioxidative defense system, disturb energy metabolism, and cause neuronal apoptosis [14,15,16].

Emerging evidence suggests that micro-RNAs (miRNAs) are associated and localized to mitochondria, indicating that the intricacy of the regulation of mitochondrial function is mediated by proteins which encoded by the nuclear and mitochondrial genomes [17,18,19]. Because mitochondria contain proteins mainly encoded by the nuclear genome [20], they may serve as a potential site for miRNA-mediated posttranscriptional regulation [21,22]. For instance, miRNAs that regulate mitochondrial function at the posttranscriptional level affect mitochondrial homeostasis, energy metabolism, oxidative stress, and apoptosis under physiological and pathological conditions [17,23,24].

The pathogenesis of cerebral ischemia-reperfusion injury involves miRNAs that alter the mitochondrial response and regulate the expression of key elements that mediate neuronal survival and apoptosis [25,26,27,28], including changes in mitochondrial membrane potential, ROS generation under oxidative stress, and apoptosis induced by energy metabolism. In this review, we briefly summarize the molecular mechanisms that determine mitochondrial function and the roles of miRNAs during ischemia-reperfusion.

## 2. Mitochondria in Ischemia-Reperfusion Injury

Mitochondria are found in almost all eukaryotic cells and function as “power houses” [29]. The mitochondrial matrix is surrounded by a permeable outer membrane and a much less permeable inner membrane. The outer membrane is freely permeable to small metabolites due to the presence of an abundant protein, and the inner membrane includes components of the respiratory chain that catalyzes oxidative phosphorylation (OxPhos). Mitochondria are the main organelles that generate adenosine triphosphate (ATP) [30] and serve as the major sites of the oxidative metabolism of carbohydrates, fats, amino acids, and other biological molecules [31,32]. Mitochondria represent a key role in the pathogenesis of cerebral ischemia-reperfusion injury, which involves ROS generation, dysfunctional energy metabolism, and mitochondria-induced apoptosis.

### 2.1. Generation of ROS

Mitochondria are potential major intracellular sources of ROS in almost all cells. The electron transport chain localized to the mitochondrial inner membrane is the main site for ROS generation [33]. Specifically, mitochondrial components such as the growth factor adaptor Shc, NADPH oxidase-4 (NOX4), and the mitochondrial redox carriers complexes I and III [34] promote production of ROS [35,36,37,38,39,40]. Under normal physiological conditions, xanthine oxidase, and the electron transport chain of the mitochondrion, arachidonic acid, and NADPH oxidase are involved in the generation of the sources of ROS, for instance, superoxide anion (·O_2_^−^), hydrogen peroxide (H_2_O_2_), and hydroxide radical (·OH^−^) [31]. Further, a series of intracellular antioxidants, for instance, superoxide dismutase (SOD), glutathione peroxidase (GSHPx), and glutathione rapidly remove excess ROS [41]. ROS remains at low levels under physiological conditions, and the homeostasis of cellular redox is crucial for numerous biological processes [42,43]. During brain ischemia-reperfusion, several processes, including the initial change in mitochondrial membrane potential and calcium overload, occur concurrently and cause neurons to undergo apoptosis. Among these processes, oxidative stress caused by excess ROS generation plays a major role in brain damage [44,45].

Sanderson *et al.* (2013) considered that mitochondrial changes during brain ischemia-reperfusion injury involve the following steps: ischemic starvation, reperfusion-induced hyperactivation, mitochondrial dysfunction, and delayed neuronal death [46]. During oxidation associated with mitochondrial respiration, energy is produced by the electrochemical potential stored in the inner mitochondrial membrane, which causes an asymmetric distribution of protons and other ions with different concentrations on both sides of the mitochondrial membrane that generates the mitochondrial membrane potential (ΔΨm). The ΔΨm is considered a reliable indicator of mitochondrial membrane permeability and a sensitive indicator of mitochondrial function [47].

Furthermore, intracellular Ca^2+^ maintains homeostasis through its uptake and release via the mitochondrial calcium uniporter [48,49], mitochondrial Na^+^/Ca^2+^ exchanger, mitochondrial permeability transition pore (MPTP), and pathways including the Na^+^-independent pathway of Ca^2+^ efflux and H^+^/Ca^2+^ antiporter pathway, *etc.* [50]. Under physiological conditions, ΔΨm is negative in the inner chamber; by contrast, it is positive in the outer chamber; only a small number of electrons “leak” in complexes I and III [42]. During ischemia, the initial change in mitochondria involves their membrane potential, and ROS generation is partly dependent on this change [39]. Insufficient oxygen delivery during ischemia causes increased electron leakage mediated by complexes I and III and triggers the disruption of ion pump function. Consequently, intracellular Ca^2+^ overload is detected, the sustained opening of the MPTP is stimulated [51], and the collapse of the electrochemical potential of H^+^ is triggered, all of which inhibits ROS production. When blood supply is reestablished, as it occurs due to membrane instability during early reperfusion, the reintroduction of oxygen enhances electron leakage, which markedly increases ROS production (called the ROS “burst”) [46]. Ca^2+^ inhibits complex I, which increases ROS generation *in vitro* and *in vivo* [52].

### 2.2. Energy Metabolism

Mitochondria generate ATP for most cells through oxidative phosphorylation to produce more than 95% of a cell’s energy under physiological conditions via complexes I–V [53]. In almost all animal models of stroke, the oxidative metabolism in the ischemic core of brain is impaired by glucose and oxygen deficiency, which rapidly alters ATP and other energy-related metabolites that are mainly involved with the mitochondria [54]. In the mitochondrial matrix, the pyruvate oxidation, fatty acid (FA) oxidation, glutamine metabolism, and tricarboxylic acid (TCA) cycle pathways are associated with energy metabolism [32]. In cerebral ischemia-reperfusion injury, energy metabolism in the mitochondria may change through various mechanisms.

Under pathological conditions, most cells in the penumbra remain viable after 2 h of ischemia; however, glucose and ATP contents decrease considerably and the phosphocreatine content decreases to approximately 70% of non-ischemic values [55]. A portion of the adenosine diphosphate (ADP) generated through ATP hydrolysis is metabolized to adenosine monophosphate and ATP. Ischemia inhibits oxidative metabolism. In contrast, anaerobic glycolysis increases indirectly and produces large amounts of lactic acid that decrease intracellular pH, causing a decrease in or loss of activities of multiple intracellular enzymes [56]. Moreover, the decrease in glucose metabolism may cause increased pyruvate oxidation [57], affecting the activation of acetyl coenzyme A and causes persistent activation of the TCA cycle [58]. Reperfusion partially restores blood flow in the brain and the glucose utilization is decreased to approximately half of the normal range in the ischemic core [59,60]. It also causes the concentration of ATP to recover more slowly than that of phosphocreatine or the adenylate energy charge. However, neurons in the penumbral tissue undergo entire or nearly entire recovery of phosphocreatine. The adenine nucleotide balance shows that the penumbra region of energy metabolism is moderately affected [60].

### 2.3. Release of Cyt c

Cyt c, which plays a major role in apoptosis, is located in the inner membrane of mitochondria, is the first proapoptotic protein discovered in the mitochondria [61]. Its function is required for OxPhos and is implicated in intrinsic type II apoptosis [62]. MPTP likely promotes apoptosis caused by Cyt c release, then activates caspases (see below) [51,63,64]. The release of Cyt c from the mitochondria into the cytoplasm is the main inducer of apoptosis [65]. The mechanisms of mitochondrial apoptosis are as follows: First, loosely coupled or tightly bound Cyt c is damaged in the mitochondrial membrane and then released. Second, Bax (see below) in the outer mitochondrial membrane increases the permeability of the outer membrane of mitochondria and stimulates the release of free Cyt c [66].

The Bcl-2 family and its regulation of mitochondrial permeability were the focus of extensive investigations. The members of the Bcl-2 family can be categorized according to their specific functions during apoptosis [67] as follows: (1) anti-apoptotic proteins, for instance, Bcl-2, Bcl-xl, and Bcl-W, which contain a minimum of three BH domains; and (2) proapoptotic proteins such as Bax, and Bak, which contain domains of BH1, BH2, and BH3. BH3-only proteins, which include Bid, Bad, and NOXA, are another group of pro-apoptotic proteins [68,69,70]. Bcl-2 family proteins contain 20 amino acid residues in the hydrophobic region in the C-terminal region that mediates interaction with the outer mitochondrial membrane. This region is present on the mitochondrial membrane and regulates the release of Cyt c. Anti-apoptotic Bcl-2 proteins, which are distributed mainly in the outer mitochondrial membrane, nuclear membrane, and endoplasmic reticulum (ER), elicit a stable organelle membrane effect. Unlike anti-apoptotic proteins, Bax and Bak directly interact with mitochondria, resulting in the release of Cyt c to trigger apoptosis [71,72].

The mitochondria involved in brain ischemia-reperfusion injury trigger apoptosis [72]. The production of ROS and release of apoptotic factors into the cytoplasm are implicated in the activation of cell death cascades. These factors mainly includes Cyt c and Apoptosis-inducing factor (AIF) [73]. Cyt c is transported through specific pores formed by proapoptotic proteins such as Bax, which are embedded in the outer mitochondrial membrane. During ischemia-reperfusion, the sequence of apoptotic events begins with the binding of Cyt c to apoptosis protein-associated factor 1 (Apaf-1) [74,75]. The Apaf-1/caspase-9/Cyt c complex is formed, and caspase-3 is activated, which triggers neuronal death [76].

## 3. miRNAs Regulate Mitochondrial Function during Cerebral Ischemia-Reperfusion Injury

miRNAs (20–22 nucleotides) regulate the expression of genes by binding to the 3′ untranslated regions of mRNAs, causing downregulation of gene expression or mRNA degradation [77,78]. The biogenesis of all miRNA families converts the primary (pri)-miRNA transcript into the active mature miRNA. The pri-miRNA transcript is cleaved by the enzyme Drosha, yielding the precursor (pre)-miRNA [79]. In the cytoplasm, pre-miRNAs are converted into 18–22 bp double-stranded RNAs [80]. After Dicer cleavage, the mature miRNA is passed into the RNA-induced silencing complex, where it guides the complex to target mRNAs [81]. Generally, passive-strand miRNA (miRNA*) is degraded and does not affect gene expression. However, recent research indicated that miRNA* molecules have similar biological effects as mature miRNAs by repressing target mRNAs [82]. Since the discovery of lin-4 and let-7, numerous miRNAs were detected using techniques such as northern blotting, deep sequencing, and bioinformatics [83,84,85]. Evidence indicates that miRNAs are essential for cellular processes such as proliferation, differentiation, and apoptosis [86,87,88].

The expression of more than 30% of genes for coding proteins are regulated by miRNAs [89]. Moreover, cerebral miRNAs are altered after ischemia-reperfusion injury, suggesting that miRNA-mediated translational may play a pivotal role in modulating gene expression [28,90]. Because the mitochondrial outer membrane is freely permeable to small metabolites, it is the main location for assembling miRNA and its processing components. miRNAs may also translocate to the mitochondrial matrix to perform their function by targeting mitochondrial genomes. Mitochondrion-associated miRNAs participate in cellular processes such as response to stress, metabolism, and death. Therefore, mitochondrial function during cerebral ischemia-reperfusion injury requires highly coordinated gene expression, which is partly modulated by numerous miRNAs.

### 3.1. Mitochondrial Oxidative Stress

**Figure 1:**
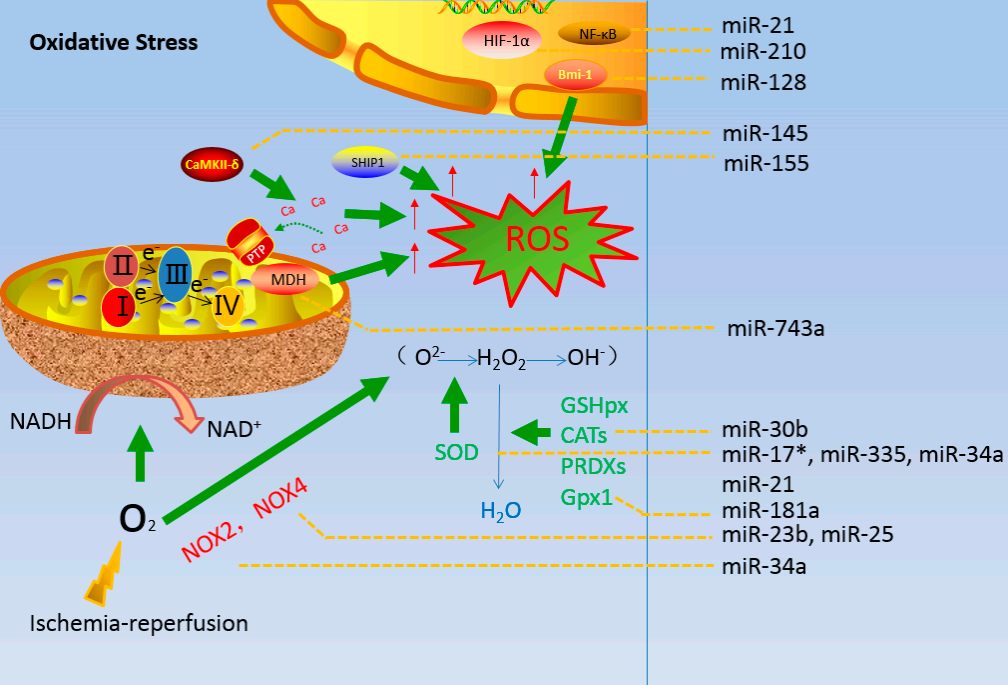
Regulatory role of miRNAs in mitochondrial oxidative stress during brain ischemia-reperfusion injury.

miRNAs have been detected within or in association with mitochondria from various tissues and cell types involved in a variety of biological processes [91,92,93]. These miRNAs function as critical regulatory molecules in the regulation of cellular redox reactions. Therefore, we assumed that these molecules act as regulators during cerebral ischemia-reperfusion injury (Table 1, lines 1 to 14). During early ischemia, the mitochondrial membrane potential changes due to hypoxia. For instance, miR-210 is stimulated by HIF-1α-regulated hypoxia and miR-210 is strongly induced in response to hypoxia, thereby activating ROS generation [94] (Figure 1). During reperfusion, there is a burst of ROS, including ·O_2_^−^, H_2_O_2_, and ·OH^−^. Moreover, miRNAs regulate ROS generation to a certain extent under conditions of mitochondrial oxidative stress. H_2_O_2_-induced upregulation of MDH is likely mediated by the downregulation of miR-743a [95]. miR-128a directly target Bim-1 to increase intracellular ROS levels and then alters the intracellular redox state [96]. miR-145 represses CaMKII-δ expression and ROS-induced Ca^2+^ overload [97]. Similarly, miR-155 represses the expression of src homology 2 (SH2)-containing inositol 5-phosphatase 1 (SHIP1) and increases ROS generation [98].

miRNAs influence the balance of ROS. For instance, NOX4 and NOX2 belong to the NOX family of NADPH oxidases that produce large amounts of ROS [99]. NOX4 was discovered in nonphagocytic cell types and tissues, and its mRNA is the direct target of miR-23b [100] (Figure 1). miRNA-25 increases the expression of NOX4 and mediates oxidative/nitrative stress as well as the consequent mitochondrial dysfunctions [101]. NOX2 increases ROS generation in various cell types, and NOX2 mRNA is the target of miR-34a, which exhibits proapoptotic activity, mainly by enhancing NOX2 expression and ROS production [102]. In addition, the ROS defense system is composed of several enzymes, including SOD, catalases (CATs), GSHPx, and PRDXs [103].

Under conditions of pathological reperfusion, three antioxidant enzymes, MnSOD, GPX2, and Txnrd2, present in mitochondria, are negatively regulated by miR-17* [104]. Moreover, the senescence of cells is inhibited by miR-335 and miR-34a through the upregulation of SOD2 and Txnrd2 expression [105] (Figure 1), causing a decrease in ROS production. Inhibition of miR-181a expression protects cells against oxidative stress-induced apoptosis through the direct inhibition of Gpx1 expression and ROS generation [106]. These antioxidant enzymes, which are the major components of the primary antioxidant system, coordinately remove the ROS generated in the mitochondria. Nevertheless, the conversion of ·O_2_^−^ to H_2_O_2_ is inhibited by miR-21, which occurs when miR-21 directly decreases SOD3 levels or indirectly reduces SOD2 levels, and ROS levels are affected by miR-21 [107]. Furthermore, NF-κB increases miR-21 expression under oxidative stress; however, H_2_O_2_-induced ROS activity is partly protected by the overexpression of miR-21 [108]. ROS is similarly regulated by miR-30b that targets CATs, and antisense molecules enhance cytoprotective mechanisms against oxidative stress by strengthening the antioxidant defense system [109].

Excessive amounts of ROS including ·O_2_^−^, H_2_O_2_, and hydroxyl radicals (OH·), are produced in the mitochondria during brain ischemia-reperfusion injury. The nuclear transcription factors HIF-1α and NF-κB contribute to the generation of ROS during cerebral ischemia-reperfusion. SOD detoxifies O_2_^−^ by converting it to H_2_O_2_, which is converted to H_2_O by CATs or GSHPx/Gpx1. NOX4 and NOX2 generate ·O_2_^−^-generating enzymes. miRNAs targeting mitochondrial-related proteins and antioxidative enzymes as well as nuclear transcription factors under mitochondrial oxidative stress are shown in Figure 1.

**Table 1:** miRNAs associated with mitochondrial oxidative stress, energy metabolism and apoptosis.

miRNAs	Target	Function	References
miR-181a	GPx1	reduces ROS production	[106]
miR-210	ISCU, COX10	activates the generation of ROS	[94]
miR-21	SOD3, TNF-α	modulates the levels of ROS	[107]
miR-743a	mdh2	increase MDH production	[95]
miR-145	CaMKIIδ	regulates ROS-induced Ca^2+^ overload	[97]
miR-155	SHIP1	enhances ROS production	[98]
miR-23b	NOX4	increases the level of ROS scavengers	[110]
miR-25	NOX4	mediates oxidative/nitrative stress	[101]
miR-34a	NOX2	enhanced ROS production	[102]
miR-30b	CATs	againsts oxidative stress	[109]
miR-17*	MnSOD, GPX2, TrxR2	clears up the high levers of ROS	[104]
miR-335, miR-34a	SOD2, Txnrd2	decrease generation of ROS	[105]
miR-128	Bmi-1	increases Intracellular ROS levels	[96]
miR-302	E2F3	reduces intracellular ROS	[111]
miR-210	ISCU, COX10	up-regulates the glycolysis	[94]
miR-23a/b	c-Myc	enhances glutamine metabolism	[112]
miR-378/378*	PGC-1β	energy metabolism	[113]
miR-378*	PGC-1β	inhibits TCA gene expression	[114]
miR-15a	UCP-2	inhibits the synthesis of insulin	[115]
miR-15b	Arl2	decrease mitochondrial integrity	[116]
miR-338	COXIV	decreases oxidative phosphorylation	[117]
miR-141	SlC25A3	influences mitochondrial ATP production	[118]
miR-199a-5p	CAV1	inhibits ATP levels, mitochondrial DNA	[119]
miR-696	PGC-1α	up-regulates aerobic metabolism	[120]
miR-122	PKM2	increases glycolysis	[121]
miR-221/222	PUMA	inhibits mitochondrial pathway of apoptosis	[122]
miR-155	p53	accumulates DNA damage	[98]
miR-134	Bcl-2	anti-apoptotic gene Bcl-2	[123]
miR-29a	BH3-only	reduces neuronal vulnerability	[124]
miR-145	BH3-only	against mitochondria apoptotic pathway	[125]
miR-30a	LC3	enhances beclin 1-mediated autophagy	[126]
miR-181a	GRP78	regulates GRP78 expression	[127]
miR-497/302b	Bcl-2	induces neuronal apoptosis	[128]
miR-21	Bcl-2	decreases Bax/Bcl-2 ratio	[129]
miR-29c	Birc2, Bak1	increases apoptosis	[130]
miR-23a	XIAP	leads to different ways of cell death	[131]
miR-23a	APAF-1/caspase-9	increases in the activation of caspase-9	[132]
miR-499	Drp1	regulates mitochondrial dynamics	[133]
miR-133	caspase-9, -3	increases caspases-9 and -3	[134]
miR-124	Ku70	against I/R-induced neuronal death	[135]
miR-761	MFF	inhibits mitochondrial fission and apoptosis	[136]
miR-214	NCX1	against Ca^2+^ overload injury and cell death	[137]

### 3.2. Energy Metabolism in Mitochondria

**Figure 2:**
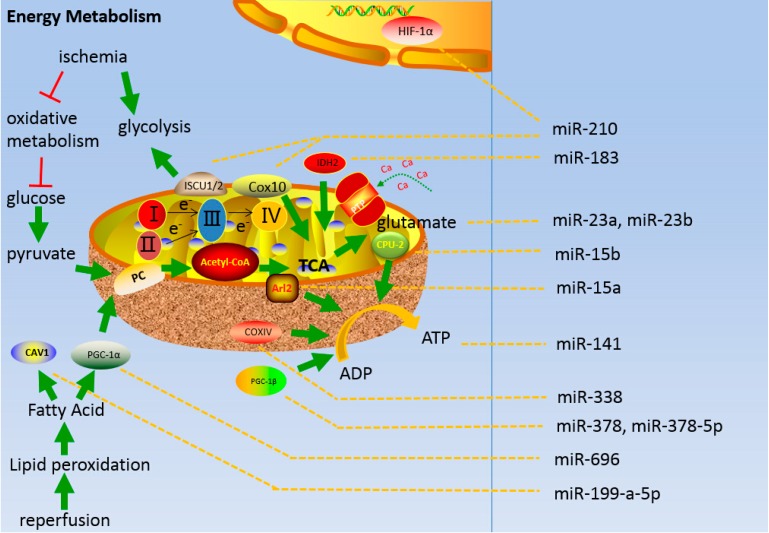
Energy metabolism in the mitochondria during cerebral ischemia-reperfusion injury and the regulatory role of miRNAs.

Mitochondrial energy metabolism during brain ischemia-reperfusion injury is regulated by miRNAs [138]. (Table 1, lines 15 to 25). During the early stages of ischemia, oxidative metabolism declines as glycolysis increases, and studies of miR-210 under hypoxia in different cell types show that the main components of hypoxic response are regulated by HIF-1α [139]. HIF-1α contributes to the metabolic shift by downregulating several steps of mitochondrial metabolism through direct inhibition of the Fe-S cluster assembly protein 1/2 and Cyt c oxidase 10 (COX10) expression, which decreases mitochondrial function and upregulates glycolysis [94] (Figure 2).

During hypoxia, glucose, ATP, and phosphocreatine levels are reduced, and miRNAs regulate ATP generation and conversion to ADP. For instance, miR-15b modulates the concentration of ATP by targeting Arl2, and miR-15b overexpression reduces Arl2 expression, and therefore inhibits ADP/ATP exchange and ATP synthesis [116]. Furthermore, miR-15a directly inhibits the expression of the gene encoding uncoupling protein-2 (UCP-2) to increase oxygen consumption and reduce ATP generation [115]. COXIV, a key protein in the electron transfer chain of the mitochondria, takes part in ATP production. The changes of COXIV levels affects mitochondrial function. Moreover, miR-338 regulates the expression of COXIV and targets a wide variety of mitochondrial mRNAs that encode vital proteins are involved in oxidative phosphorylation [117].

miR-378 inhibits PGC-1β expression [113], whereas miR-378-5p activates PGC-1β expression and induces reduction in tricarboxylic acid cycle gene expression [114] (Figure 2). Numerous miRNAs function during reperfusion which involves in lipid peroxidation and excess lactate production with excessive ROS generation and synthesis of glutathione from glutamate [140]; the following are the examples: miR-23a/b represses glutaminase expression [112], miR199-a-5p participates in mitochondrial activity and mitochondrial β-oxidation. miR-199-a-5p overexpression exacerbates the deposition of FA and decreases ATP and mitochondrial DNA (mtDNA) concentrations by inhibiting caveolin1 [119]. miR-696 directly targets the activated receptor gamma co-activator 1-α (PGC-1α) and regulates FA oxidation capacity and mitochondrial biogenesis. Overexpression of miR-696 decreases FA oxidation and mtDNA content [120]. miR-141 regulates SLC25A3 expression, and affects mitochondrial ATP production [118]. The overexpression of miR-183 inhibits the expression of IDH2, which is one of the mitochondrial enzymes related to the TCA cycle [141].

During cerebral ischemia, oxidative metabolism and glucose content decrease considerably, but glycolysis is sustained, causing impaired oxidative metabolism of pyruvate by mitochondria. The process of generating energy by glucose oxidative metabolism mainly includes the generation of pyruvate, pyruvate metabolism, and TCA cycle. Lipid peroxidation occurs during reperfusion. The miRNAs involved in this process are indicated on the right.

### 3.3. Mitochondrial Pathways of Apoptosis

**Figure 3:**
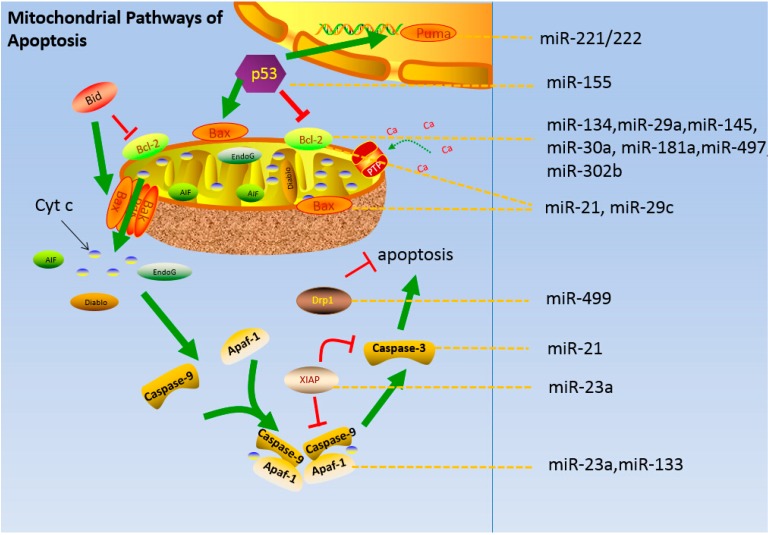
Apoptosis under pathological conditions of reperfusion and the regulatory roles of miRNAs.

Mitochondria-induced neuronal apoptosis during ischemia-reperfusion involves reduction of the mitochondrial membrane potential and opening of the MPTP that is associated with Bcl-2 family proteins [142]. Cyt c, AIF, and other apoptotic factors are released into the cytoplasm, as a result, Apaf-1 is activated, the caspase cascade is stimulated, and cell apoptosis is directly induced [143,144]. miRNAs involved in the mitochondrial apoptosis pathway regulate various proteins (Table 1 lines 26 to 42). For instance, p53 regulates the cell cycle and apoptosis, and p53 is activated in a microglia subpopulation in the inflamed human brain, causing cell death triggered via the mitochondrial apoptosis pathway [145,146] (Figure 3). p53 negatively regulates c-Maf in microglia by inducing miR-155 [147]. However, p53 activation and pernicious cascade resulting in the mitochondrial apoptotic pathway are limited by miR-30a [148].

The mitochondrial apoptotic pathway is inhibited by miR-221/222 through the regulation of the BH3-only protein PUMA [122]. Bnip3, a member of the BH3-only protein family, is a direct target of miR-145 and regulates the mitochondrial apoptotic pathway during myocardial ischemia-reperfusion injury [125]. miR-29a is highly expressed in astrocytes, and it targets PUMA to decrease ischemic injuries *in vitro* and *in vivo* and reduces ischemic brain injury by attenuating neuronal vulnerability [124]. Moreover, miR-29a targets multiple Bcl-2 family members, including those that are proapoptotic or anti-apoptotic, to regulate cell proliferation [149,150]. miR-181 levels change in response to stroke; in addition, increased GRP78 levels are implicated in cellular functions such as protein folding in the ER and inhibiting apoptosis [127] and in increasing the expression of Bcl-2 family members. Furthermore, global cerebral ischemia and focal ischemia are alleviated by inhibiting miR-181a production [151].

Moreover, Bcl-2 is a target of miR-134, miR-497, and miR-302 [123,128,152]. The overexpression of either miR-497 or miR-302b reduces the expression of their target genes and increases caspase-3-mediated apoptosis [128]. The downregulation of miR-134 reduces ischemic injury by enhancing the expression of CREB [123] (Figure 3). Moreover, miRNAs strongly regulate the downstream steps in the mitochondrial apoptotic pathway, and antisense miR-23a enhances apoptosis via the APAF-1/caspase-9 apoptotic pathway [132]. The X-linked inhibitor of apoptosis (XIAP), which is the main endogenous caspase inhibitor, is the target of miR-23a. After a stroke, miR-23a expression in men and women differ significantly, leading to caspase-dependent and -independent cell deaths in women and men, respectively [131]. miR-133 increases caspase-9 and caspase-3, thereby stimulating the mitochondrial apoptotic pathway [134] and miR-21-induces decreases in the Bax-to-Bcl-2 ratio and caspase-3 activity [129]. miRNA-29c directly targets the mRNAs encoding Birc2 and Bak1 and increases apoptosis [130]. Mitochondrial fission occurs through phosphorylation and dephosphorylation of dynamin-related protein 1 [153], which is regulated by miR-499 [133].

Cerebral ischemia-reperfusion injury triggers the mitochondrial release of Cyt c, AIF, Diablo, and EndoG via Bak. Cyt c then binds to procaspase-9 and Apaf-1 to form the apoptosome. The procaspase-9 complex is transactivated to activate caspase-9. Caspase-9 cleaves and activates downstream caspases such as caspase-3 to induce apoptosis. XIAP simultaneously inhibits caspases-9 and caspase-3. Bcl-2 and Bax prevent Cyt c release and interfere with this pathway. p53 and PUMA are involved in this process. miRNAs that target the mitochondrial apoptotic pathways are indicated on the right in Figure 3.

## 4. Perspectives

Stroke is a multi-factor disease, with limited therapeutic strategies. Therefore, numerous clinical trials to treat this disease have failed [154]. The early stage of cerebral ischemia is accompanied by hypoxia, and mitochondria are the most sensitive organelles to hypoxia. During cerebral reperfusion, changes in mitochondria may partly predict disease progression. The levels of some miRNAs change significantly in cerebral ischemia-reperfusion injury [155,156]. In addition, miRNAs target various mitochondrial and mitochondria-associated proteins. Therefore, miRNAs may serve as useful tools to manipulate mitochondrial function (Table 1). The pathological process of the ischemia-reperfusion cascade in the brain involves a complex series of events, which may be driven by multiple cellular pathways that act coordinately, and miRNAs simultaneously regulate numerous target genes [157]. Therefore, miRNAs are potential targets for the treatment of stroke (Table 2). Moreover, products of one miRNA target genes may belong to the same functional protein-protein interaction network [158]. miRNAs may perform a range of functions, including regulation of long noncoding RNAs [159] and control of epigenetic mechanisms [160]. Current research indicates that the mechanisms of miRNA-targeting therapeutics include the following: (1) change in the absorption, distribution, metabolism, and excretion of anti-miRNAs; (2) upregulate the expression of targeted miRNAs or enhance their biogenesis; and (3) upregulate other miRNAs that target the same genes [161]. Most miRNA-targeting molecules of stroke are in the preclinical stage, and certain obstacles to their use must be removed. Although the multiplicity of binding targets presents potential difficulties, the modulation of miRNA levels may provide a new strategy for treating stroke and represents a potentially effective treatment paradigm.

**Table 2:** miRNAs as therapeutic targets.

miRNAs	Indications	Method	Developmental Stage	References
miR-181	cerebral ischemia	antagomir therapy	preclinical	[127]
miR-181	cerebral ischemia	antagomir therapy	preclinical	[162]
miR-181b	ischemic stroke	antagomir therapy	preclinical	[163]
miR-497	ischemic brain injury	antagomir therapy	preclinical	[152]
Let7f	ischemic stroke	antagomir therapy	preclinical	[164]
miR-424	cerebral I/R	antagomir therapy	preclinical	[165]
miR-200c	cerebral ischemia	antagomir therapy	preclinical	[166]

## Abbreviations

ATP: adenosine triphosphate; Apaf-1: apoptosis protein-associated factor 1; Cyt c: cytochrome c; Drp1: dynam-in-related protein 1; ER: endoplasmic reticulum; FA: fatty acid; MCU: mitochondrial calcium uniporter; miRNA: microRNA; MPTP: mitochondrial permeability transition pore; Nox4: NADPH oxidase-4; OxPhos: oxidative phosphorylation; ROS: reactive oxygen species; TCA: tricarboxylic acid; UCP-2: uncoupling protein-2; ΔΨm: mitochondrial membrane potential.
